# The Association Between Test Anxiety, Self-Efficacy, and Mental Images Among University Students: Results From an Online Survey

**DOI:** 10.3389/fpsyt.2021.618108

**Published:** 2021-11-30

**Authors:** Anna Maier, Caroline Schaitz, Julia Kröner, Alexander Berger, Ferdinand Keller, Petra Beschoner, Bernhard Connemann, Zrinka Sosic-Vasic

**Affiliations:** ^1^Department for Psychiatry and Psychotherapy, University Clinic of Ulm, Ulm, Germany; ^2^Devision for Applied Psychotherapy, Hospital Christophsbad Göppingen, Göppingen, Germany; ^3^Department for Child and Adolescent Psychiatry and Psychotherapy, University Clinic of Ulm, Ulm, Germany; ^4^Department for Psychosomatic Medicine and Psychotherapy, University Clinic of Ulm, Ulm, Germany

**Keywords:** test anxiety, anxiety, self-efficacy, mental images, imagery

## Abstract

**Background and Objectives:** A substantial portion of students report test anxiety, and those reporting low levels of self-efficacy seem to be especially affected. Previous research has indicated the relevance of mental images in the maintenance of anxiety disorders, however, no data are available with respect to test anxiety. In order to close this gap, the present study investigates the association between test anxiety, self-efficacy and mental images.

**Method:** One hundred sixty-three university students completed an online survey. Test anxiety (PAF), general self-efficacy (WIRKALL-r), study-related self-efficacy (WIRK_STUD), intrusiveness of mental images (IFES), spontaneous use of imagery (SUIS) and vividness of imagery (VVIQ) were examined.

**Results:** Test-related mental images were frequently reported among the surveyed students. Test anxiety showed a positive correlation with IFES and a negative correlation with self-efficacy. Mediation analyses showed that about one fifth of the influence of self-efficacy on test anxiety is mediated by IFES.

**Discussion:** The present study gives first indication about an association between test anxiety, self-efficacy and mental images, even though the results are limited with respect to generalizability. Further investigations with respect to the impact of test-related mental images on the self-efficacy/test-anxiety linkage are needed.

## Introduction

Test anxiety is a common phenomenon among pupils, college students, and university students, with occurrence rates between 20 and 40% being reported among students (1, 2). Test anxiety is expressed by cognitive, affective, physiological and behavioral levels (3). Negatively distorted perceptions of the exam, its relevance, and its outcome are commonly reported with associated difficulties in concentration. Those affected by test anxiety typically report feelings of despair, hopelessness and failure, but also panic-like physiological reactions such as accelerated heart rate, bladder and intestinal activation, sweating, and nausea. Avoidance of exam situations, or at least, rumination about avoiding future exams can be observed among individuals suffering from test anxiety. Notwithstanding the frequency of the occurrence of test anxiety and the psychological burden for those affected, test anxiety is not listed as a diagnostic entity within current classification frameworks. Neither ICD-10 (International Classification of Mental Disorders) (4) nor DSM 5 (Diagnostical and Statistical Manual of Mental Disorders) (5) list test anxiety as a separate diagnosis. In clinical practice, symptoms of test anxiety are mostly diagnosed as a specific phobia (with respect to test anxiety) or social phobia (with respect to fear of performance evaluation) (1). This complicates the definition of test anxiety and leads to an inconsistent use of the term in the literature (3). Nonetheless, research has produced some models of test anxiety (6). Cognitive and emotional dimensions (7) are assumed. The cognitive component mainly includes worries, which are inversely related to performance expectations, while no connection could be shown between emotionality and performance expectations. The frequently used questionnaire to record test anxiety (PAF) is also based on a model with various factors. Here, too, cognitive factors such as worries and emotional factors such as excitement are recorded (8). Recent studies also try to take social factors into account. There are initial indications that there are cultural and gender-specific differences for individual subscales (e.g. Hyperarousal, Worries). (9). Beside the psychological burden, a typical short term impairment of test anxiety is its negative impact on learning motivation and learning behavior, whereas its long term impairment includes a negative impact on performance outcomes (9, 10). Among those, test anxiety is assumed to be associated with reduced working memory performance (11) and unproductive cognitive processing (12). In a recent study by Cassady and colleagues (6), some influencing factors on the extent of test anxiety could already be extracted. Fear was higher when the outcome of the task was uncertain, passive learning strategies were used, or learning strategies were more personal involved. For many other anxiety disorders mental images associated with the feared situation or object are well-described (13). Mental images are vivid perceptual information that occur in all sensory modalities and are perceived as real experiences (14). Frequently, retrospective images arise from memory about past events [like reliving past stressful events as seen in PTSD (15)]. Recent research also indicates the occurrence of prospective images that are associated with situations in the future not yet experienced, but in the case of anxiety disorders, feared. Hirsch and Holmes gave an overview (13) about the role of mental images within anxiety disorders. In summary, mental images are present in almost all anxiety disorders, but in different ways. In PTSD, the imageries occur in the form of flashbacks regarding the traumatic experience (16), whereas in agoraphobia, the imageries are related to a situation where it is impossible to deal with a physical or mental crisis (17, 18). In social phobia the aversive imagery occurs primarily in the form of their own mistakes or embarrassing situations (19). It turned out that perspective also plays an important role in this context. In a study of Hackman and colleagues (20), participants with social phobia were more likely to have external imageries and had experiences with “seeing themselves through others eyes” (p. 3).

For test anxiety, first insights about its association with mental images are emerging. In their online survey of over two hundred German-speaking students, Klug and colleagues identified that around one third of the investigated individuals suffered from test-related intrusions. The amount of intrusions further increased exponentially with the amount of test anxiety, in particular, when visual intrusions became present (21). Further evidence for a possible relationship between mental images and test anxiety stems from three imagery-based intervention studies [including two randomized controlled trials (RCTs)]. All of them observed the efficacy of a new treatment approach applying Imagery Rescripting (IR) in self-reported test anxiety (22–24). In this procedure, unpleasant mental images are altered in the imagination with the guidance of the therapist. The result is an imagination with modified emotions and more positive experiences of coping (25). Since test anxiety could be reduced by modifying mental images, it is reasonable to assume a connection between the two constructs.

Another well-investigated influencing factor on test anxiety is self-efficacy. This concept is defined as “people's judgments of their capabilities to organize and execute courses of action required to attain designated types of performances” (26). The study of self-efficacy began in the 1970s when Albert Bandura (27, 28) mentioned it as part of his social cognitive learning theory. It has received more research than many other comparable models (29). Self-efficacy is a predictor for various behavioral outcomes such as social skills, athletic performances, or coping with feared events (26). Self-efficacy also seems to play an important role in learning-relevant aspects. In general, self-efficacy and academic performance are substantially related to each other as confirmed by a meta-analysis (30, 31) wherein 14% of the variance of the academic performance could be predicted by self-efficacy (32). Furthermore, a high correlation between self-efficacy and intrinsic motivation has been reported (30, 31). In relation to this, students with high self-efficacy are better in self-monitoring within the learning context (33). Another review came to a similar conclusion. 59 studies examining self-efficacy and performance in an academic context were specifically evaluated. In addition to the expected correlation between self-efficacy and academic performance, moderating influencing factors such as effort regulation, deep processing strategies and goal orientations were found. This illustrates the importance of self-efficacy for learning and performance again (34).

With respect to test anxiety and self-efficacy, several studies have reported an association between both of these variables [e.g. (35)]. If learners believe in their own abilities for the task at hand, motivation increases and good performance is more likely (35). On the other hand, the less likely the person concerned the success and their own competencies, the lower the motivation (36). In a study by Onyeinzugbo (37), a regression analysis showed that self-efficacy contributed to 14% of variability in test anxiety, while gender was not a significant predictor. It was confirmed that high test anxiety is associated with lower self-efficacy. Similar results were found based on a structural equation model investigating the fear of mathematics as a function of self-concept (38). The model indicated that, regardless of gender, a high degree of perceived self-efficacy was negatively associated with test anxiety (38). In line with this, a meta-analysis confirmed a negative association between self-efficacy and test anxiety. Also, there were negative consequences of test anxiety on motivation, behavior, and cognition (39). Furthermore, other studies showed that the study outcome in terms of grades could be predicted based on test anxiety and self-efficacy, with self-efficacy acting as a moderating variable between test anxiety and the grade (40).

In contrast, thus far the association between self-efficacy and mental images has been investigated only in sports psychology. Mills, Munroe, and colleagues (41) concluded that competitive athletes with higher self-efficacy used more motivating imaginations than athletes with lower self-efficacy. A study in novice climbers also showed that positive imagery is associated with lower levels of stress and higher self-efficacy (42). Furthermore, a meta-analysis showed the benefits of practicing mental imagery on motor skills performance (43).

**Figure 1:**
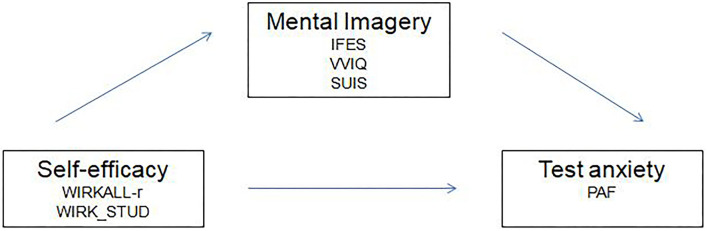
Mediation model.

However, despite this first indication of a relevant association between test anxiety and mental images on the one hand, and fairly robust findings on an association between test anxiety and self-efficacy on the other hand, to date, there are no studies on the interaction between test anxiety, self-efficacy, and mental images. Notwithstanding, it seems reasonable to assume a possible mediation. First, imagery rescripting, as defined above, has proven to be effective in decreasing test anxiety. The application of such imagery-based techniques enable the individual to experience a certain capability to cope with seemingly hopeless situations by replacing dysfunctional beliefs and actions with more functional ones during the course of imagination. This intervention is very likely to evoke experiences of self-efficacy, which then might have an impact on test anxiety. As a consequence, it could be assumed that mental images influence the connection between self-efficacy and test anxiety, and act as a mediation variable. Thus, the present study addresses the following questions:

1. Do test-related mental images occur within test anxiety, and if so, are there differences with respect to the occurrence during examination periods and outside examinations periods among students suffering from test anxiety?
2. Can the finding of a negative connection between test anxiety and self-efficacy be replicated?
3. Do mental images mediate the influence of self-efficacy on test anxiety? (Figure 1).

## Materials and Methods

### Participants

The study was addressed to pupils or students with subjective test anxiety. The sample was recruited via flyers and public announcements at the local university, social media groups (Facebook), e-mail-lists, and the homepage of the conducting department of the University Clinic of Ulm, Germany. The study was advertised as a survey about test anxiety and excessive excitement before exams, and it was intended to target students who have had experiences with these. Individuals interested in participating in the study were able to follow a link to the questionnaire, presented via the software Enterprise Feedback Suite (EFS) Survey (44). For motivational reasons, an expense allowance of 3 × 100 € was raffled among the participants. No additional financial compensation was given. In total, 163 university students (78.5% female) completed the online survey. Inclusion criteria were frequent or recurrent experiences of test anxiety, age over 18 years, a present university student or pupil status, and German as their native language.

#### Study Flow

In total, 338 people used the link to the study. Of these, 105 people did not start the study and another 70 discontinued the survey. The remaining 163 participants completed the survey and only the completers were used for the evaluation.

#### Use of Assistance With Test Anxiety

As can be seen in Table 1, the majority of participants (65%) had never used any form of assistance with respect to test anxiety. When reporting the utilization of assistance in dealing with their test anxiety, reading self-help guides (25.2%) was most frequently reported. The next most frequent help that was utilized was the attendance of counseling centers (6.7%) directly followed by attendance of psychotherapeutic treatment (4.9%). About 6.7% of the subjects used other forms of assistance in dealing with test anxiety: Friends (3%), homoeopathic and herbal remedies (1.8%), learning groups (0.6%), and the use of mental strategies (0.6%) were also mentioned. Participants who reported that they have never used any kind of assistance were able to report the respective reasons for not doing so. Among the participants who indicated reasons, eight reported perceiving themselves as good at dealing with anxiety by personal strategies such as sports, good preparation, or faith. Seven reported that they did not have enough information about test anxiety assistance offers in the past. Six indicated that they wanted to deal with their test anxiety on their own, while six other subjects reported feelings of shame that prevented them to seek help. Other reasons were lack of time, no motivation, unwillingness to admit the test anxiety, or considering test anxiety as a normal phenomenon occurring in most students making professional help unnecessary. Furthermore, some participants reported that while experiencing test anxiety, their symptoms were debilitating in a way such that they were unable to seek counseling or any other form of help.

**Table 1:** Frequencies and percentage of help service utilization with respect to test anxiety.

**Assistance**	**Frequency (%)**
None	106 (65 %)
Self-help guides	41 (25.2 %)
Counseling center	11 (6.7 %)
Other	11 (6.7 %)
Psychotherapist	8 (4.9 %)
Family doctor	4 (2.5 %)
Psychiatrist	1 (0.6 %)

### Design

The study was operationalized as a cross-sectional online survey. The participants were able to participate in the study from home and from any mobile internet-enabled device. Prior approval from the local ethics board (University of Ulm, Germany) was obtained.

### Procedure

The investigation was presented with the software “Enterprise Feedback Suite” EFS Survey (44). Potential participants had to follow a given link in order to participate the study. Subsequent to this, a window with study information and conditions for participation appeared. Afterwards, participants had to confirm the informed consent. For help or further questions a contact address was presented. Participants were forwarded to the questionnaire after providing consent. The questionnaire contained some open-ended questions with text fields for answers, while others were presented as single or multiple-choice questions to click on. Furthermore, validated self-rating scales (see 2.5.2 for more details) with a Likert rating to click on were included in the survey questionnaire. For participants' orientation, their status of questionnaire completion was evident throughout, as a graphic display clarified the percentage spread. The processing of the online questionnaire took 19 min on average.

### Measures

#### Socio-Demographic Data

Information on gender, age, nationality, and marital status, as well as the use of health care services with respect to test anxiety were collected. In addition, the number of years of education, their upcoming exam preparation, and the duration until the upcoming exam were recorded. The answer format consisted of open answer options via text field and predefined categories with single- or multiple-choice options.

#### Test Anxiety

Test anxiety was assessed by the German test anxiety questionnaire *Prüfungsangstfragebogen* [*PAF*, (8)] consisting of 20 items. Individual differences in the level of habitual test anxiety (agitation, anxiety, interference, and deficiency of confidence) were captured. Questions were asked about different thoughts and feelings during test anxiety among a 5-point Likert scale. Below are some sample items for the respective subscales: (a) Agitation: “The heart beats up to my neck.”, (b) Worriedness: “I think of what happens if I do badly.”, (c) Interference: “I forget things because I'm too busy with myself.”, and (d) Deficiency of confidence: “I trust my performance.” (positive coded). The questionnaire has a high internal consistency (Cronbach's α = 0.88) and retest reliability (*r* = 0.86) (8).

#### Self-Efficacy

The general self-efficacy was estimated using the 10-item German questionnaire, *Fragebogen zur allgemeinen Selbstwirksamkeit* [*WIRKALL_r*, (45)]. A sample item is “If there is resistance, I find ways and means to assert myself.” The WIRKALL_r has high internal validity (Cronbach's α = 0.86) (45). Study-specific self-efficacy was assessed by the 7-item German questionnaire *Fragebogen zur studiumsspezifischen Selbstwirksamkeit* [*WIRK_STUD*, (46)]. A sample item for WIRK_STUD is “Even if an exam is difficult, I am sure that I will do well.” This questionnaire has also a satisfactory internal consistency (Cronbach's α = 0.77) (46). Both questionnaires are answered on a 4-point Likert scale.

#### Mental Imagery

The German version of the *Vividness of Visual Imagery Questionnaire* [*VVIQ*, (47)] was used to scale the individual distinctions in vividness of visual imagery. Participants were instructed to imagine four situations (e.g. “Imagine a rising sun. Look carefully at the image that appears in your mind's eye. The sky brightens and surrounds the sun with blue color.”) and answer the vividness of the imagery on a 5-point Likert scale (perfectly clear and as vivid as normal vision, clear and reasonably vivid, moderately clear and vivid, vague and dim, no image at all, and you only “know” that you are thinking of the object). The questionnaire has a high reliability, ranging from Cronbach's α of 0.88 or 0.96 when using the split-half method (48). In addition, the German and adapted translation of the 24-item *Impact of Future Events Scale* [*IFES*, (49, 50)] was assigned. Participants were asked to respond to each item with respect to recently (i.e. last seven days) experienced test anxiety. The IFES measures on a scale from 0 (not at all) to 4 (very strong) just how frequent prospective, i.e. future-related, mental images regarding upcoming exam situations associated with test anxiety occur, and how frequent re-experiencing (e.g. “Pictures about the exam popped into my mind.”), hyperarousal (e.g. “I had waves of strong feelings about the exam.”) and avoidance (e.g. “I tried not to think about the exam.”) associated with the test-related mental images were experienced in the last week. The IFES has high alpha indices (Cronbach's α =0.87) and a good test-retest reliability of r = 0.73 (51). Furthermore, the German version of the *Spontaneous Use of Imagery Scale* [*SUIS*, (52)] was employed to record via 18 items the interindividual differences in the daily occurrence of imagery (e.g. “If I go to a place that is unknown to me, I would rather have directions that contain, in addition to the names of landmarks, detailed descriptions of them (such as the size, outline and color of a gas station”)) on a 5-level Likert scale. The SUIS demonstrated good internal consistency (Cronbach's α = 0.81) and a high convergent validity (52). Moreover, the seven self-developed questions below about the presence, type, frequency during and outside exam periods, valence, the level of anxiety in the worst moment of the imagination, and perspective of the imaginations were integrated: “Do you know any visual ideas related to test anxiety?” (Yes – No); “Future imaginations, imaginations directed toward future events, can be distinguished from past imaginations, imaginations directed toward past events. Which of these types do you know?” (Only future imaginations - Only past imagination – Both types); “How often do these imaginations generally occur during the exam period?”; “How often do these impressions generally occur outside of the exam period?” (Response scale for both items: Always - Often - Sometimes – Never); “How high is your fear at the worst moment of imagination on a scale of 0-100 (0 = no fear, 100 = highest possible fear)?”; “How do you usually feel about the pictures?” (Very unpleasant - Rather unpleasant - Neutral - Rather pleasant - Very pleasant.); “Please remember an exam situation in which you felt anxious and uncomfortable. It can be a written or oral exam or a situation in the preparatory phase where you feel fear. Once you find such a situation, imagine it as clearly and clearly as possible. If you want, you can close your eyes. What perspective do you take? Do you see the situation as if through your own eyes or rather from an outside perspective?” (Observer/external perspective - I see as through my own eyes – Other).

#### Academic Success

To register the study performance, two self-developed items were added: “How do you rate your own academic achievements/your academic success?” and “Please enter your current average grade (take into account the achievements of the last examination period).” German grades between one (highest possible grade) and six (lowest possible grade) were allowed.

### Statistical Analysis

The data were analyzed using the statistics program SPSS Statistics Version 25 [International Business Machines Corporation (53) for Microsoft and R (54)]. Descriptive data such as relative and absolute frequencies, means, medians, and standard deviations were used to describe the sampling characteristics, the use of assistance, and the characteristics of imaginations. There was also a qualitative evaluation of the reasons given for the lack of use of professional help. Furthermore, correlations were calculated using the Pearson correlation. The alpha error was α = 0.05 for the calculations. In addition, mediation models were calculated to investigate a mediating effect of mental imagery on the influence of self-efficacy and on anxiety. Study-related self-efficacy (WIRK_STUD) was chosen as self-efficacy measure for these mediation models, as it showed a larger correlation with test anxiety compared to general self-efficacy (see section 3.3). We calculated mediation models for each of the three mental imagery scales (VVIQ, SUIS, IFES). Mediation models were calculated using the R package “mediation” (55). Confidence intervals and p-values were obtained using a bootstrap procedure with 1000 simulations.

## Results

### Occurrence and Characteristics of Mental Images

In total, 55.8% of the entire sample reported adverse mental images associated with test anxiety. Nearly all mental images were neither exclusively related to past or future exams. Instead, they occurred in a mixed form containing pictures from memory as well as parts reflecting exam situations never experienced before. About 3.1% reported mental images of only past or future events. Among those participants reporting test-related mental images, 38.5% experienced themselves from an external perspective when confronted with the mental image, while 39.6% saw the image from the first-person-perspective or through their own eyes. Furthermore, 20.1% of the participants were familiar with both of these perspectives while one person reported to perceive the image from the perspective of an examiner. The differences in the appearance of mental images during and outside the exam period are shown in Table 2. On average, the mental image at its most distressing moment was rated as adverse with a score of 7 (SD = 2.09) on a scale from 0 to 10. About 85.7% rated the imageries as very or rather uncomfortable, 11% as neutral and 3.3% as very or rather pleasant. The people who rated neutral or pleasant imageries were excluded from relevant analyses because it can be assumed that they have already presented helpful coping situations in the context of mental images and were, therefore, not the target group. Hence, *N* = 150 subjects remained for the following analyses.

**Table 2:** Appearance of mental images during and outside the exam time.

	**During exam period**	**Outside exam period**
Always	16 (9.8 %)	6 (3.7 %)
Often	30 (18.4 %)	23 (14.1 %)
Sometimes	43 (26.4 %)	42 (25.8 %)
Never	2 (1.2 %)	20 (12.3 %)

### Test Anxiety, Mental Imagery and Self-Efficacy

Test anxiety as assessed by the mean PAF score was related to several aspects of mental imagery. There was a strong positive correlation between test anxiety and the intrusiveness of mental images related to future exams as assessed by the mean IFES score (*r* = 0.509, *p* < 0.001), and a moderate positive correlation between test anxiety and the spontaneous use of imagery (SUIS) (*r* = 0.228; *p* = 0.005). Also, there was a moderate positive correlation between test anxiety and the frequency of occurrence of test-related images during (*r* = 0.441, *p* < 0.001) and outside exam periods (*r* = 0.258, *p* = 0.013), as well as between the test anxiety and the degree of anxiety in the worst moment of imagination (*r* = 0.475, *p* < 0.001). Furthermore, there was a moderate negative correlation between test anxiety and the valence of the mental image (*r* = −0.389, *p* < 0.001), with higher valance values indicating more comfort during the image.

General self-efficacy was recorded via WIRKALL-r, and study specific self-efficacy using WIRK_STUD. In general, test anxiety was moderately to strongly negatively correlated with general self-efficacy and study-related self-efficacy, respectively (*r* = −0.454, *p* < 0.001; *r* = −0.628, *p* < 0.001).

### Mediation Analyses

We investigated mediating influences of the three mental imagery scales (VVIQ, SUIS, IFES) on the relation of study-related self-efficacy (WIRK_STUD) and test anxiety (PAF). The reported coefficients are standardized estimates. The intrusiveness of mental images significantly mediated the influence of study-related self-efficacy on test anxiety (*p* < 0.001). 21.4% of the effect of WIRK_STUD on PAF could be explained by IFES (95%-CI = 11–34%). The direct effect of WIRK_STUD on PAF was β = −0.496 (*p* < 0.001, 95%-CI: −0.62–−0.37), while the effect of WIRK_STUD on IFES was β = −0.379 (*p* < 0.001, 95%-CI: −0.53–−0.23) and the influence of IFES on PAF was β = 0.355 (*p* < 0.001, 95%-CI: 0.23–0.48). The average causal mediation effect, i.e. the influence of WIRK_STUD on PAF which was mediated by IFES, was β = −0.135 (*p* < 0.001, 95%-CI: −0.21–−0.07). To sum up, higher study-related self-efficacy was associated with lower test anxiety and about a fifth of this effect could be explained by lowered intrusiveness of mental images. Neither VVIQ (*p* = 0.89) nor SUIS (*p* = 0.74) mediated the influence of WIRK_STUD on PAF.

## Discussion

The present study is among the first to explore test-related mental images within university students experiencing test anxiety. The leading research focus was placed on the interaction between test-related mental images and test-anxiety, as well as self-efficacy and test anxiety. In addition, the assumed mediating influence of mental images on the effect of self-efficacy on test anxiety was also evaluated.

Help-seeking behavior among test anxious students was determined. Interestingly, more than half of the participants had never sought help with respect to their test anxiety, and if so, self-help was the most frequent approach taken. Several reasons were identified, summarized as (a) access barriers such as insufficient information about potential assistance and (b) personal barriers such as shame or lack of time for time-costly interventions. These findings are in line with the very few data available thus far. Blanco and colleagues (56) reported in their large national epidemiological study that only 20% of college students suffering from anxiety disorders sought professional help, while similar reasons to those reported in the present study were identified. These results might reflect a preference amongst students with anxiety disorders for low-threshold services such as brief psychotherapeutic interventions in the treatment of their shame-associated anxiety and could be indicative for the need of more dissemination of such interventions.

Further, we found prevalent imaginations in the context of test anxiety. In our sample, more than half of the participants reported aversive mental images, mostly with respect to future situations. Images referring to past situations were reported less frequently. Furthermore, in accordance with other studies (57), we found different perspectives of mental images. However, in contrast to findings on social phobia (20), the present work did not show any higher occurrence of an observer perspective. This is surprising as in clinical practice, symptoms of test anxiety are mostly diagnosed as social phobia (with respect to fear of performance evaluation) (1). A possible explanation for the obtained balanced distribution of observer- and first-person perspectives could be the occurrence of both, retrospective as well as prospective imaginations. According to Berntsen & Rubin (58), more distant memories tend to occur from an observer's perspective whereas more recent memories from a first-person perspective. In addition, imaginations in the first-person perspective turned out to be more emotional, as they are associated with higher degrees of affective reactions and psychophysiological states (59). This may suppose that prospective images are more stressful and induce a higher level of anxiety than retrospective ones. In general, our results revealed that test-related mental images are perceived as aversive and unpleasant. This result seems plausible considering that emotional images are experienced as particularly vivid as compared to neutral ones (60). Furthermore, such images often become indistinct with reality and a clear separation of imagination and reality by the person concerned is no longer possible (61, 62).

One major finding of the present study was a significant association between test anxiety and mental images. This association was substantial regardless of the time period such mental images typically appeared, i.e. within or outside the stressful examination phases, even though test-related mental images occurred more often during examination times. Furthermore, and in accordance with the results of Klug et al. (21), test anxiety was associated with higher intrusiveness of the mental images as assessed by IFES and there was a general higher occurrence of the imaginations within participants reporting higher test anxiety values. In line with this, the fear during the worst moment of the imagination correlated positively with test anxiety. Accordingly, the valence of the images was rated as more uncomfortable in cases where test anxiety was higher. These results, similar to the aforementioned study of Klug and colleagues, confirm an association between test anxiety and mental images, which is already known for other anxiety disorders and mental images (21, 63). In addition, we were able to show that test anxiety can be predicted by imagery. In particular, IFES accounts for substantial variance clarification, which supports the assumption of more test anxiety in prospective images. These results also suggest that test anxious people with negative mental images are possibly more burdened. Similar conclusions were drawn from other studies on several other mental disorders. Holmes et al. (64) summarized a more instable mood in patients with bipolar disorder when imaginations are reported. This is obvious as mental images are known to operate as an “emotional amplifier” (65). Furthermore, a study by Borcovec & Inz (66) uncovered the strain that mental images pose. Back in 1990, they assumed that worrying in terms of verbal (rather than imaginal) concerns in generalized anxiety disorder presents a strategy for inhibiting mental images, and thus avoiding strong emotional states. Therefore, it can be deduced how important research on mental images is, especially with regard to patient care. If the psychological strain in test-anxious people with imaginations is greater, the suffering may be reduced more effectively by considering such imaginations. Some studies of short interventions with Imagery Rescripting (22–24, 67) have already shown first evidence for the effectiveness of such interventions, and the present results provide theoretical background for test anxiety and mental images.

Second, according to our hypotheses, we were able to replicate the well-known negative relationship between test anxiety and self-efficacy (37, 68). It was shown that study-specific self-efficacy proved to be a good predictor of test anxiety and confirmed the relevance of study-related self-efficacy: the more self-efficacy, the lower the test anxiety. This result is not surprising as test anxiety occurs mainly in the context of studying. Third, we analyzed various possible mediating imagination variables between self-efficacy and test-anxiety. We found a significant mediation-effect for the variable IFES. That means the well-known association between higher study-related self-efficacy and lower test anxiety could be explained by lowered impact of mental images. The present study was the first examining this connection. Nonetheless, there have been studies in the past that suggest an influence of mental imagery on the relationship between self-efficacy and test anxiety (13, 37). In accordance with our results, earlier studies (65, 66) suggest that greater vividness and impact of mental images evoke greater emotional involvement. It is therefore obvious that when both factors occur, the fear increases more. However, in the present study, no mediating effects for further imagination variables (SUIS, VVIQ) were shown, although several variables of imagination and self-efficacy were included. There are several possible explanations: Either (a) imagery *per se* does not act as mediator, which in turn may be indicative for a parallel but independent effect of self-efficacy and imagery on test anxiety, or (b) imaginations operate as a mediator, but only in terms of their impact. In latter case, it could be likely that vividness and the spontaneous use of imaginations act a minor part. However, even though our present significant result for the impact of future events gives a first indication of a possible mediating effect of mental images, this result must be interpreted with caution due to the limitations described below.

## Limitations

The present study has several limitations which need to be considered. First, since only university students were included in the present survey, generalizability of the present results is limited. It is disputable whether the findings may be applied to other groups of people with test anxiety, such as school children or trainees. In addition, generalizability is limited due to the large proportion of female participants. Even though the gender proportion of the present study is representative for test anxiety where females in general are twice as much affected than males (69), one cannot rule out that male sufferers from test anxiety were less addressed in this sample. Another limitation is the average test anxiety (PAF) *T*-value of 58, which is just below the cut-off for surpassing test anxiety. Increasing the cut-off to 60 would have indicated that the value was at least one standard deviation above average (8). Since this value was not exceeded in the present sample, this may have limited the results. Therefore, it seems interesting to repeat the analysis in another study with more pronounced test anxiety scores.

## Conclusion

The present study is one of the first to investigate the occurrence of mental images in the context of test anxiety among university students. We were able to identify several characteristics associated with such mental imagery: Test-related imageries present themselves intrusively, are experienced aversively, and occur predominantly with respect to future examination situations and occur from a first-person perspective. Furthermore, test-related mental images seem to predict the burden of test anxiety. In addition, this study is the first to investigate the connection between self-efficacy and test anxiety with respect to imaginations via mediation analysis. The observed mediation of self-efficacy, intrusiveness of mental images and test anxiety provides first indications for the relationship between these variables and may be indicative for future research. Against the background of the increasing evidence of the effectiveness of imagery-based interventions in the treatment of anxiety disorders, further research appears essential for a fundamental grasp of the mechanism behind these approaches. Future studies could further investigate the impact and mediating effect of test-related mental images on the course of illness and therapy outcome within test anxiety.

## Data Availability Statement

The raw data supporting the conclusions of this article will be made available by the authors, without undue reservation.

## Ethics Statement

The studies involving human participants were reviewed and approved by Ethikkommission der Universität Ulm. The patients/participants provided their written informed consent to participate in this study.

## Author Contributions

AM: study conception and design, analysis and interpretation of data, and manuscript writing and editing. CS, JK, PB, and BC: study design and manuscript editing. AB and FK: data analysis and interpretation of data and manuscript editing. ZS-V: study conception and design, interpretation of data, manuscript editing, and critical revision. All authors contributed to the article and approved the submitted version.

## Conflict of Interest

The authors declare that the research was conducted in the absence of any commercial or financial relationships that could be construed as a potential conflict of interest.

## Publisher's Note

All claims expressed in this article are solely those of the authors and do not necessarily represent those of their affiliated organizations, or those of the publisher, the editors and the reviewers. Any product that may be evaluated in this article, or claim that may be made by its manufacturer, is not guaranteed or endorsed by the publisher.
